# Use of carbon monoxide and hydrogen by a bacteria–animal symbiosis from seagrass sediments

**DOI:** 10.1111/1462-2920.12912

**Published:** 2015-07-23

**Authors:** Manuel Kleiner, Cecilia Wentrup, Thomas Holler, Gaute Lavik, Jens Harder, Christian Lott, Sten Littmann, Marcel M. M. Kuypers, Nicole Dubilier

**Affiliations:** ^1^ Max Planck Institute for Marine MicrobiologyCelsiusstrasse 1Bremen28359Germany; ^2^ Department of GeoscienceUniversity of Calgary2500 University DriveCalgaryABT2N 1N4Canada; ^3^ Department of Microbiology and Ecosystem ScienceDivision of Microbial EcologyUniversity of ViennaAlthanstr. 14A‐1090ViennaAustria; ^4^ Elba Field StationHYDRA Institute for Marine SciencesVia del Forno 80Campo nell'ElbaLI57034Italy

## Abstract

The gutless marine worm *O* *lavius algarvensis* lives in symbiosis with chemosynthetic bacteria that provide nutrition by fixing carbon dioxide (CO_2_) into biomass using reduced sulfur compounds as energy sources. A recent metaproteomic analysis of the *O* *. algarvensis* symbiosis indicated that carbon monoxide (CO) and hydrogen (H_2_) might also be used as energy sources.

We provide direct evidence that the *O* *. algarvensis* symbiosis consumes CO and H_2_. Single cell imaging using nanoscale secondary ion mass spectrometry revealed that one of the symbionts, the γ3‐symbiont, uses the energy from CO oxidation to fix CO_2_. Pore water analysis revealed considerable *in‐situ* concentrations of CO and H_2_ in the *O* *. algarvensis* environment, Mediterranean seagrass sediments. Pore water H_2_ concentrations (89–2147 nM) were up to two orders of magnitude higher than in seawater, and up to 36‐fold higher than previously known from shallow‐water marine sediments. Pore water CO concentrations (17–51 nM) were twice as high as in the overlying seawater (no literature data from other shallow‐water sediments are available for comparison). *Ex‐situ* incubation experiments showed that dead seagrass rhizomes produced large amounts of CO. CO production from decaying plant material could thus be a significant energy source for microbial primary production in seagrass sediments.

## Introduction

Mutualistic symbioses between bacteria and animals are widespread, occur in almost all animal phyla and play major roles in the development, health and evolution of their hosts (McFall‐Ngai, 2002; Walker and Crossman, 2007; Moya *et al*., 2008; Fraune and Bosch, 2010; McFall‐Ngai *et al*., 2013). In many mutualistic symbioses, the function of the bacterial symbionts is to provide essential nutrients to their hosts (Moran, 2007; Moya *et al*., 2008). In chemosynthetic symbioses, the bacteria provide all or most of their host's nutrition using inorganic compounds such as sulfide or hydrogen (H_2_) as energy sources to fix carbon dioxide (CO_2_) into biomass (Stewart *et al*., 2005; DeChaine and Cavanaugh, 2006; Dubilier *et al*., 2008; Petersen *et al*., 2011; Kleiner *et al*., 2012a).

**Figure 1:**
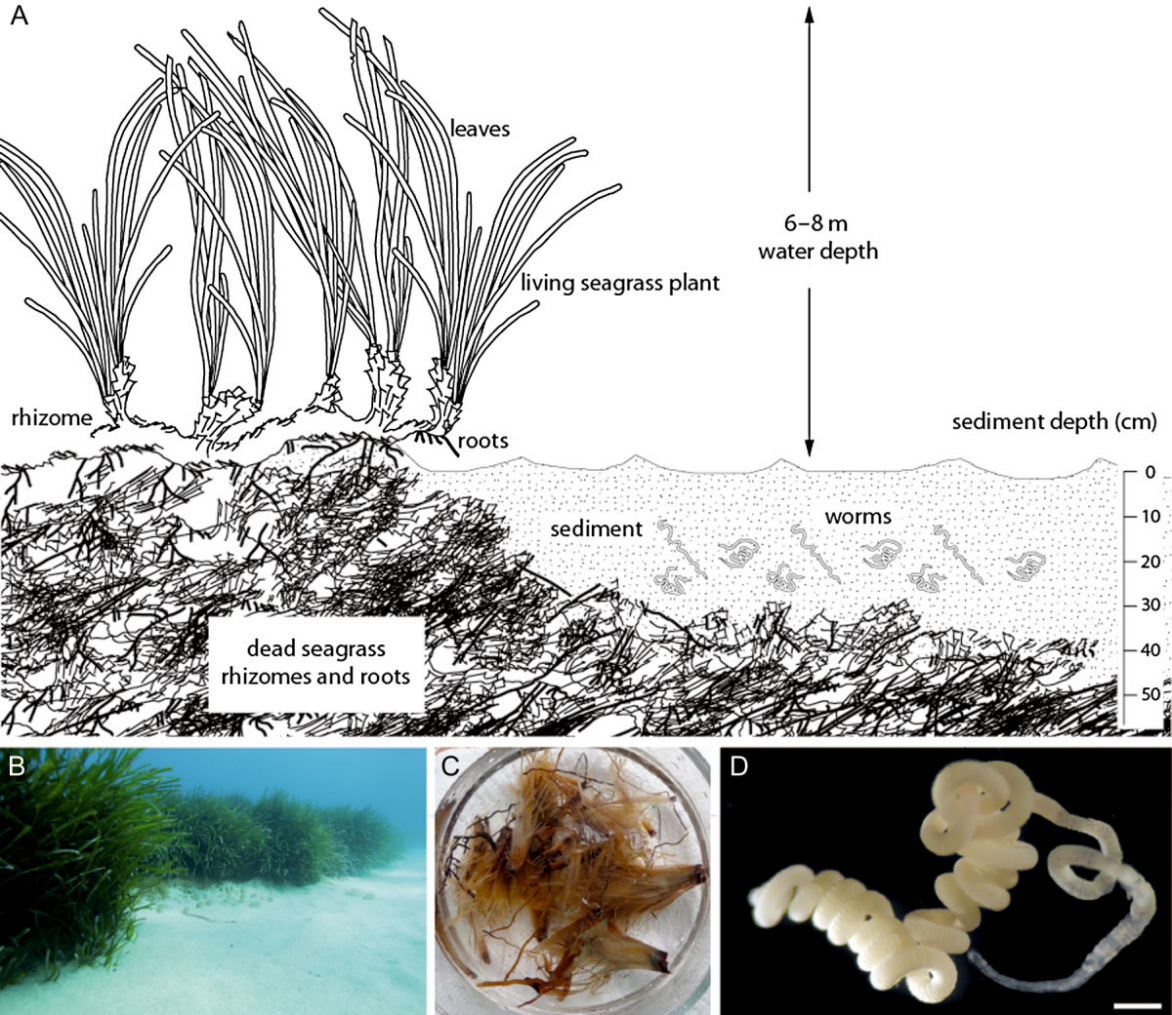
*O* *lavius algarvensis* and the Mediterranean seagrass sediments it inhabits. A. The *O* *. algarvensis* environment is characterized by medium‐ to coarse‐grained silicate sediments and patches of the seagrass *P* *osidonia oceanica*. Subsurface roots and rhizomes (horizontal stems) stabilize the plants in the sediment. The roots and rhizomes form dense mats that are very stable, even after the seagrass has died, and can remain in the sediment for millennia (Mateo *et al*., 1997; Alcoverro *et al*., 2001; Duarte, 2002; Boudouresque *et al*., 2009; Gutiérrez *et al*., 2011). At the collection site for this study (Sant' Andrea in the north of the Island of Elba), reef‐like mats of dead rhizomes are buried underneath the sediment in the entire bay. The sediment overlying the rhizome mats is very poor in nutrients and energy sources (Kleiner *et al*., 2012b). B. Image of the *O* *. algarvensis* collection site showing sandy sediments surrounded by seagrass beds in 5–6 m water depth. C. Dead seagrass rhizomes from the *O* *. algarvensis* collection site. D. *Olavius algarvensis*, scale bar = 0.4 mm.

The marine oligochaete *Olavius algarvensis* does not have a digestive or excretory system and relies on its bacterial symbionts for nutrition and waste recycling (Dubilier *et al*., 2001; Giere and Erseus, 2002; Woyke *et al*., 2006; Ruehland *et al*., 2008; Kleiner *et al*., 2011; 2012b). It harbours two chemosynthetic sulfur‐oxidizing gammaproteobacterial symbionts (γ1 and γ3), two sulfate‐reducing deltaproteobacterial symbionts (δ1 and δ4) and a spirochaete between its cuticle and epidermal cells (Giere and Erseus, 2002; Ruehland *et al*., 2008). The energy sources that fuel the *O. algarvensis* symbiosis are still not well understood. The collection site for *O. algarvensis* in this study and previous studies from the same site, a shallow bay off the coast of the Island of Elba (Italy) in the Mediterranean Sea (Dubilier *et al*., 2001; Giere and Erseus, 2002; Woyke *et al*., 2006; Ruehland *et al*., 2008; Kleiner *et al*., 2012b), is characterized by *Posidonia oceanica* seagrass meadows and medium‐ to coarse‐grained sandy sediments that cover a thick, peat‐like structure consisting of dead seagrass rhizomes (Fig. 1). Concentrations of reduced sulfur compounds at this site are in the low nanomolar range, much lower than the micromolar concentrations that are usually present at sites with chemosynthetic symbioses (Dubilier *et al*., 2001; Kleiner *et al*., 2012b). In the *O. algarvensis* symbiosis, the reduced sulfur compounds required by the sulfur‐oxidizing γ‐symbionts are provided internally by the sulfate‐reducing δ‐symbionts (Dubilier *et al*., 2001). However, the external energy sources that power the symbiosis have remained enigmatic.

Metaproteomic analyses of the *O. algarvensis* association showed that three of its symbionts may use carbon monoxide (CO) and H_2_ as energy sources (Kleiner *et al*., 2012b). Both sulfate‐reducing symbionts abundantly expressed anaerobic carbon monoxide dehydrogenases (CODHs), which enable the use of CO as an energy source, as well as hydrogenases for the use of H_2_ as an energy source (Kleiner *et al*., 2012b). The third symbiont, the sulfur‐oxidizing γ3‐symbiont, abundantly expressed an aerobic CODH (Kleiner *et al*., 2012b), an enzyme used by bacteria to oxidize CO with oxygen or nitrate (King and Weber, 2007). Because the symbionts are only separated from the environment by a thin cuticle, which is highly permeable for small molecules, they are unlikely to be limited in their access to dissolved gases in the worms' environment (Dubilier *et al*., 2006).

The goal of the current study was to test if the metaproteomic predictions by Kleiner and colleagues (2012b) that CO and H_2_ are used as energy sources by the *O. algarvensis* symbiosis are correct by examining the following questions: (1) Are CO and H_2_ consumed by the *O. algarvensis* symbiosis? (2) If so, is the energy gained from CO and H_2_ oxidation used for CO_2_ fixation? (3) Are CO and H_2_ present in the *O. algarvensis* habitat, and if so what is their source and distribution?

## Results

### The *O* *. algarvensis* symbiosis oxidizes CO to CO_2_

**Figure 2:**
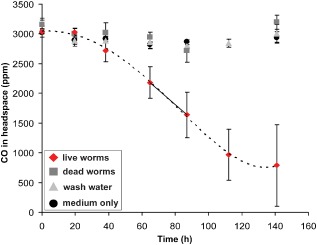
CO consumption by *O* *. algarvensis*. CO was consumed in incubations with live *O* *. algarvensis* worms, but not in controls. Consumption rates of live worms were calculated based on linear rates between 65 and 87 h (solid line). Mean values and standard deviations of three independent incubation bottles are plotted for each control and treatment.

**Figure 3:**
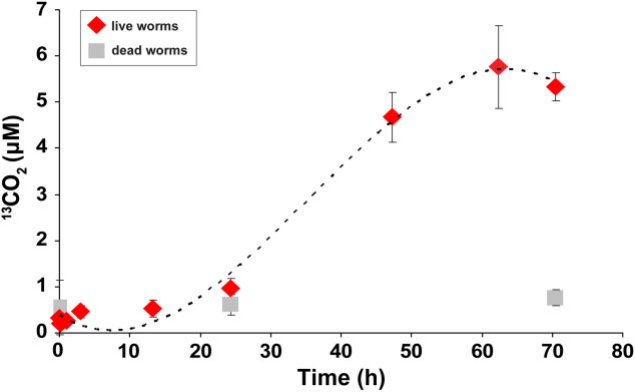
Oxidation of ^13^ CO to ^13^ CO_2_ by *O* *. algarvensis*. ^13^ CO oxidation to ^13^ CO_2_ in live and dead *O* *. algarvensis* worms was measured over 70 h after the addition of ^13^ CO (7 μM at start of incubations) and ^13^ CO_2_ was produced. Mean values and standard deviations of four independent incubation bottles are plotted for each treatment and control. Standard deviations were very small in the first four time points and are therefore not visible.

In incubation experiments, CO consumption by live *O. algarvensis* worms began after 20–40 h and CO concentrations in the headspace of incubation bottles decreased from 3040 ± 30 ppm to 790 ± 680 ppm over 141 h (Fig. 2). No notable consumption of CO was observed in controls [dead worms, water that worms were washed in and pure artificial seawater (ASW) medium] (Fig. 2). The CO consumption rate of *O. algarvensis* was 2 ± 0.5 μmol g^−1^ (wet weight) h^−1^. In incubation experiments with ^13^C‐labelled CO, *O. algarvensis* worms almost completely oxidized ^13^CO to ^13^CO_2_ within 62 h, whereas no notable production of ^13^CO_2_ occurred in the controls (dead worms) (Fig. 3). The average blank‐corrected end‐point CO concentration in the incubations with ^13^CO was 6.5 ± 35.8 nM (equivalent to 9 ± 48 ppm headspace concentration).

### The *O* *. algarvensis* symbiosis consumes H_2_

**Figure 4:**
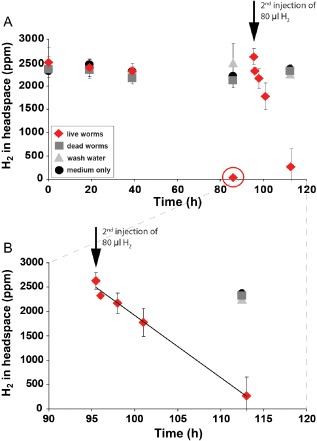
H_2_ consumption by the *O* *. algarvensis* symbiosis. A. H_2_ consumption by live worms began only after 40 h and was then completely consumed within 86 h (circled in red) in all three replicates (standard deviations at this time point were so small that they are not visible in this figure). A second injection of 80 μl of H_2_ to the incubations with live worms was monitored in shorter intervals and revealed a linear consumption of H_2_ by the *O* *. algarvensis* symbiosis. B. Close‐up of A after second H_2_ injection (at 95.5 h after begin of incubations). Linear consumption is emphasized by solid line (R ^2^ = 0.99). Mean values and standard deviations of three independent incubation bottles are plotted for each control and treatment.

H_2_ consumption by live *O. algarvensis* worms did not begin until after 40 h of incubation (Fig. 4A). After this lag phase, H_2_ consumption rates were high and H_2_ was nearly completely consumed after 86 h (from 2500 ± 320 ppm to 30 ± 20 ppm; Fig. 4A). A second injection of H_2_ into these incubations (t = 95.5 h) allowed us to better resolve H_2_ consumption over time. H_2_ decreased from 2630 ± 170 ppm to 270 ± 380 ppm within 17.5 h (Fig. 4B). The H_2_ consumption rate of the *O. algarvensis* symbiosis was 11 ± 1 μmol g^−1^ (wet weight) h^−1^. No notable consumption of H_2_ occurred in the controls (dead worms, water that worms were washed in and pure ASW medium).

### The γ3‐symbiont uses CO as an energy source to fix CO_2_ into biomass

**Figure 5:**
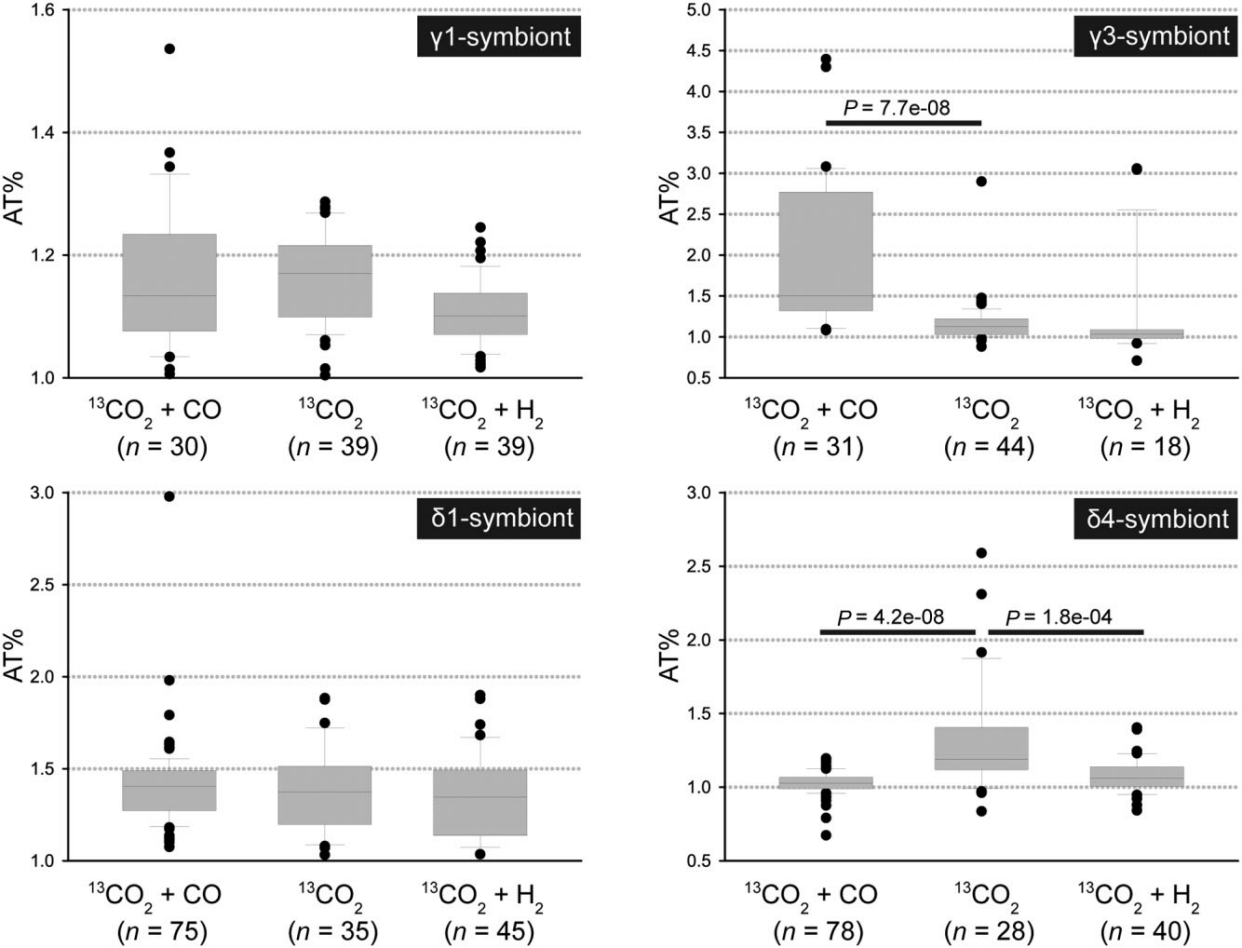
^13^C‐content of single symbiont cells based on nanoSIMS analysis. For all symbionts and treatments cells from three worms were analysed except for the γ3‐symbiont in the H_2_ treatment, for which only cells from two worms were analysed. Horizontal bars with *P*‐values indicate significant differences based on a Kruskal–Wallis test. Due to the different ^13^ C‐contents of the four symbionts we used different scales for the y‐axis for optimal visualization of the data. AT%: atom percent [^13^ C / (^12^ C + ^13^ C) × 100]; *n*: total number of symbiont cells analysed. ^13^ C isotope content values for all individual cells can be found in Table S1.

**Figure 6:**
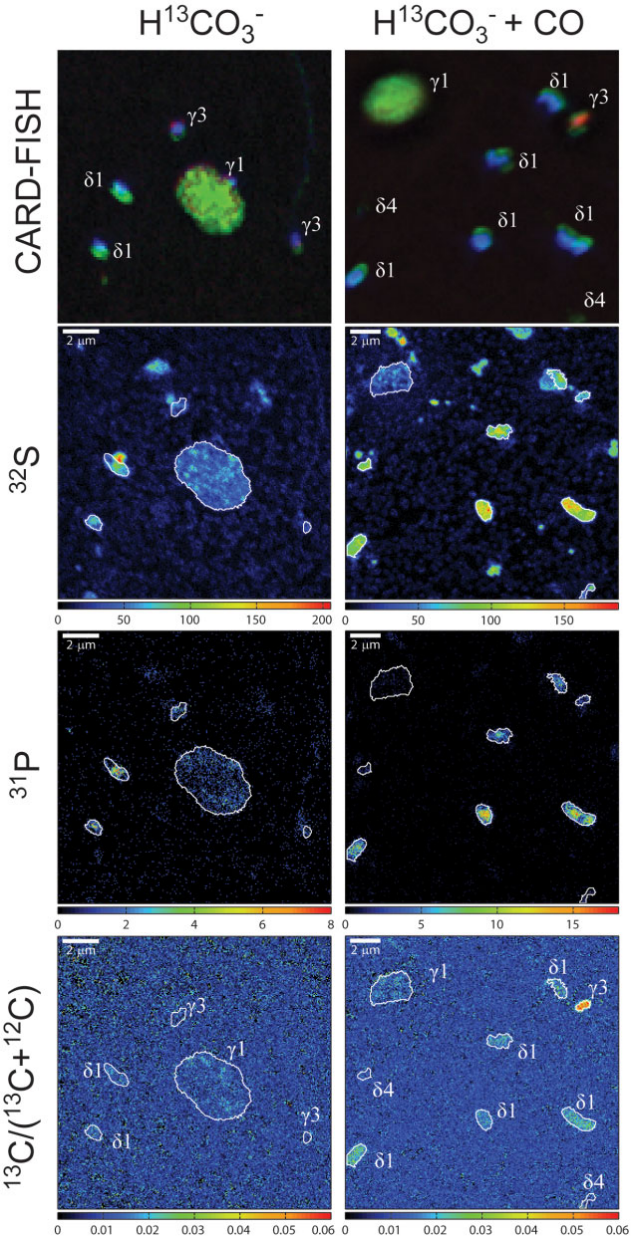
Comparison of ^13^ CO_2_ fixation by single *O* *. algarvensis* symbiont cells in the presence and absence of CO. Increased carbon fixation in the presence of CO was only visible in the γ3‐symbionts (bottom right image). Images in the left and right columns show in the top row epifluorescence micrographs of *O* *. algarvensis* symbionts on a filter, followed by the corresponding nanoSIMS images for sulfur (^32^ S ^‐^, counts per pixel) in the second row, phosphorus (^31^ P ^‐^, counts per pixel) in the third row and ^13^ C‐content as ^13^ C / (^13^ C + ^12^ C) in the bottom row. In the epifluorescence images symbiont cells hybridized with the general eubacterial probe (EUB338I‐III) are green, the sulfur‐oxidizing symbionts targeted by the gammaproteobacterial probe (Gam42a) are red and the general DNA stain 4,6‐diamidino‐2‐phenylindole (DAPI) is shown in blue. The strong green fluorescence signal of the eubacterial probe (EUBI‐III) masks the red fluorescence signal of the Gam42a probe in the γ1‐symbiont (for images showing the single channels separately see Fig. S1).

Our bulk analyses of ^13^CO_2_‐incorporation in whole *O. algarvensis* worms showed that live worms always incorporated significant amounts of ^13^CO_2_ compared with dead worms (Table 1). However, no significant differences in ^13^C‐content were detectable between live worms incubated with CO and H_2_ compared with control incubations with no experimentally added energy source (Table 1). Nanoscale secondary ion mass spectrometry (nanoSIMS) analyses of the symbionts at the single cell level revealed that in live worms all symbionts, except the δ4‐symbiont, had a higher ^13^C‐content compared with whole dead worms and the carbon signal from polycarbonate filter background (Table 1 and Table S1; Figs 5 and 6 and Fig. S1). This suggests that all symbionts except the δ4‐symbiont incorporated ^13^C under all three incubation conditions. However, because we could not measure ^13^C‐content of single symbiont cells from dead worm controls, the exact amount of ^13^C‐incorporation into individual symbionts could not be determined.

**Table 1:** ^13^ C‐content of whole worms based on bulk measurements in AT%

	Mean AT%^c^	SD AT%	Min. AT%	Max. AT%	*n*	P‐value t‐test versus dead^a^	P‐value t‐test versus w/o e^−^‐donor^b^
^13^CO_2_ + CO	1.284	0.013	1.274	1.299	3	5e‐10	0.56
^13^CO_2_ + H_2_	1.295	0.038	1.257	1.332	3	5.2e‐07	0.83
^13^CO_2_ w/o e^−^‐donor	1.303	0.048	1.26	1.355	3	2.3e‐06	–
Dead worms	1.072	0.001	1.07	1.073	6	–	–

a *P*‐values for comparisons of ^13^C‐content in worms from incubations versus dead worms (t‐test, one‐tailed, H_0_ = ^13^C‐content of live worms is not higher than that of dead worms). After Bonferroni correction for three comparisons the significance threshold *P* < 0.01 corresponds to *P* < 0.003.

b *P*‐values for comparisons of ^13^C‐content in worms incubated without additional energy source versus incubations with CO or H_2_ added (t‐test, two‐tailed, H_0_ = means are equal).

c The detailed data including the measurements on the standard caffeine can be found in Table S3.

AT%: atom percent [^13^C / (^12^C + ^13^C) × 100]. w/o e^−^‐donor: without an additional energy source added to the incubations. *n*: total number of bulk worm samples (each containing eight worms).

Analyses of single‐cell ^13^CO_2_‐incorporation in each symbiont species for significant differences between the CO and H_2_ incubations compared with control incubations with no experimentally added energy source revealed: (1) The γ3‐symbiont incorporated significantly more ^13^C‐labelled CO_2_ in the presence of CO compared with incubations without an added energy source (Kruskal–Wallis, *P* = 7.7e‐08, Fig. 5). (2) The δ4‐symbiont incorporated less ^13^C‐labelled CO_2_ in the presence of CO or H_2_ compared with incubations without an added energy source (Kruskal–Wallis, *P* = 4.2e‐08 with CO and Kruskal–Wallis, *P* = 1.8e‐04 with H_2_). (3) In the γ1‐ and δ1‐symbionts, no significant differences in ^13^C‐content in the CO and H_2_ incubations compared with control incubations with no experimentally added energy source were observed.

It is important to note that the ^13^C content of the symbionts analysed with nanoSIMS was likely diluted because of the deposition of unlabelled carbon during the catalyzed reporter deposition fluorescence *in situ* hybridization (CARD‐FISH) treatment (Musat *et al*., 2014; Woebken *et al*., 2015) potentially obscuring additional significant differences. It is also noteworthy that ^13^C‐incorporation by the symbionts can only explain part of ^13^C‐incorporation into whole worms because ^13^C‐incorporation in individual symbionts was in a similar range as in whole worms and the symbionts only make up a small fraction of the total worm biomass (Fig. 5, Table 1). The additional ^13^C‐incorporation in whole worm bulk measurements is likely due to heterotrophic CO_2_ fixation by host tissues.

### Elevated CO and H_2_ concentrations in the *O* *. algarvensis* habitat

**Figure 7:**
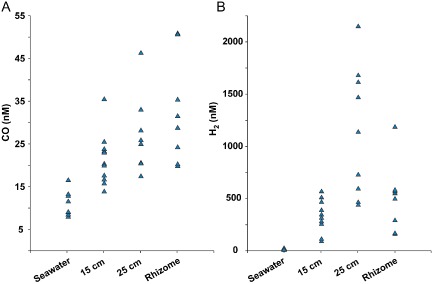
(A) CO and (B) H_2_ concentrations at the *O* *. algarvensis* collection site. Concentrations were measured at 15 and 25 cm sediment depth, in the dead rhizome mats underlying the sediment, and in the seawater about 5 cm above the sediment. For each sediment depth and control at least eight independent samples were measured. Values are blank corrected.

CO and H_2_ concentrations in sediment pore water where the worms were collected were much higher than in the seawater above the sediment (Fig. 7). Pore water CO concentrations (17–51 nM) were approximately twice as high as in seawater (8–16 nM), with the highest CO concentrations detected in pore water from within the dead rhizome mats (Figs 1 and 7). Pore water H_2_ concentrations (89–2147 nM) were up to two orders of magnitude higher than in the seawater (0–23 nM). In contrast to CO, the highest H_2_ concentrations were measured at 25 cm sediment depth and not in the deeper dead rhizome mats.

### Dead seagrass rhizomes release large amounts of CO

In incubations with dead seagrass rhizomes from the *O. algarvensis* habitat we observed high production rates of CO, whereas CO production rates in incubations with unfiltered seawater, and sediment with 0.2 μm‐filtered seawater were in the same range as in controls with double distilled water (Table 2). We measured the highest CO production rates in incubations with dead rhizomes to which ZnCl_2_ was added to stop biological activity (Table 2). Dead rhizomes produced up to two orders of magnitude more CO in incubations with ZnCl_2_ than in incubations without (3.64–55.60 versus 0.33–17.46 μmol CO kg^−1^ (dry weight rhizome material) day^−1^) (Table 2). We did not detect H_2_ production in any of the incubations with natural substrates from the *O. algarvensis* habitat.

**Table 2:** Carbon monoxide release by components from the *O* *. algarvensis* habitat and double distilled water (ddH_2_O) in μmol kg^−1^ (or l^−1^) day^−1^. ZnCl_2_ was added to incubations to stop biological activity. Mean values and standard deviations are given in parentheses

	Without ZnCl_2_	With ZnCl_2_
Dead rhizomes (dry weight)	0.33–17.46 (4.86 ± 6.95)	3.64–55.60 (24.08 ± 19.12)
Dead rhizomes (wet weight)	0.04–1.85 (0.55 ± 0.75)	0.65–6.53 (3.42 ± 2.17)
Seawater (volume)	0–0.04	0–0.01
Sediment (dry weight)	0	0
ddH_2_O (volume)	0.01–0.02	0.01–0.02

*n* ≥ 3 per incubation type.

## Discussion

### Is CO an energy source for autotrophic carbon fixation in the *O* *. algarvensis* symbiosis?

Our results indicate that part of the CO_2_ fixation in the *O. algarvensis* symbiosis is powered by the oxidation of CO by at least the γ3‐symbiont. This observation is in agreement with the abundant expression of an aerobic CODH and enzymes of the Calvin–Benson–Bassham (CBB) cycle under natural conditions in the γ3‐symbiont (Kleiner *et al*., 2012b). It suggests that the γ3‐symbiont uses its CODH (Fig. S2) to oxidize CO to CO_2_ and that it uses a part of the energy from CO oxidation to fix CO_2_ via the CBB cycle as described for other CO oxidizers (reviewed in King and Weber, 2007).

In contrast to the γ3‐symbiont, the sulfate‐reducing δ1‐ and δ4‐symbionts did not incorporate significantly more ^13^CO_2_ in the presence of CO compared with the control without CO (Fig. 5, Table S1), despite the fact that these symbionts express anaerobic CODHs under environmental conditions (Kleiner *et al*., 2012b). It is possible that the sulfate‐reducing symbionts use CO as an energy source for other metabolic functions, as known from ‘carboxydovores’, microorganisms that oxidize CO without coupling the energy gain to autotrophic growth (reviewed in King and Weber, 2007). The sulfate‐reducing symbionts could use the energy released by the oxidation of CO for lithoheterotrophic growth on organic substrates such as fatty acids that are abundantly produced by the host under anoxic conditions (Kleiner *et al*., 2012b).

Unexpectedly, the δ4‐symbiont incorporated significantly less labelled CO_2_ in the presence of CO or H_2_ compared with controls without an added energy source (Fig. 5). The δ4‐symbiont might have been inhibited by the oxygen concentrations used in this study, or by end products of the other co‐occurring symbionts. However, incubation conditions were similar in all three treatments, and ^13^CO_2_‐incorporation was clearly not inhibited in this symbiont in the control incubations without an external energy source (Fig. 5). We therefore do not have a satisfactory explanation for these results.

CO oxidation started after a lag phase of about 20 h (Fig. 2). This lag phase could be due to the CO‐free pre‐incubations of the worms (see Experimental Procedures). In aerobic CO‐oxidizing microorganisms the oxidation of CO is catalysed by an inducible CODH, which was shown to only be induced in the presence of CO (Meyer and Schlegel, 1983). It is thus likely that the symbionts did not have substantial amounts of CODH at the beginning of the incubation and had to produce it after exposure to CO.

CO oxidation rates have, to our knowledge, not been previously measured in an animal–bacterial symbiosis. In the *O. algarvensis* symbiosis, CO oxidation rates (2 ± 0.5 μmol g^−1^ (wet weight) h^−1^) were 10–100 times higher than those of bacterial CO oxidizers from coastal seawaters (0.02–0.23 μmol g^−1^ (wet weight) h^−1^) (Tolli *et al*., 2006), but comparable with rates of cultured carboxydotrophic microorganisms that can live on CO as their sole energy source (0.02 μmol to 18 mmol g^−1^ (wet weight) h^−1^) (Diekert and Thauer, 1978; Cypionka *et al*., 1980; Tolli *et al*., 2006). This comparison of CO oxidation rates has several limitations. These include the fact that CO oxidation rates for cultured CO oxidizers were determined at a range of different CO concentrations and that the mixing environment within the worm will greatly differ from a well‐shaken liquid culture.

It is likely that CO oxidation rates of the carboxydotrophic *O. algarvensis* symbionts are even higher than 2 ± 0.5 μmol g^−1^ (wet weight) h^−1^, because we estimated rates based on the biomass of the entire worm. If we assume that only the γ3‐symbiont oxidized CO, based on the observation that it was the only symbiont that incorporated significantly more ^13^CO_2_ in the presence of CO (Fig. 5), and estimate its abundance at 5% of the total biomass of whole worms (Giere and Erseus, 2002; Ruehland *et al*., 2008), CO oxidation rates would be as high as 40 μmol g^−1^ (wet weight) h^−1^. However, *in‐situ* CO oxidation rates of the *O. algarvensis* symbiosis are likely to be lower because of the lower *in‐situ* concentrations of CO compared with the concentrations used in our incubations.

### Is H_2_ also an energy source for the *O* *. algarvensis* symbiosis?

H_2_ consumption rates of the *O. algarvensis* symbiosis (11 ± 1 μmol g^−1^ (wet weight) h^−1^) were higher than those measured in the deep‐sea hydrothermal vent mussel *Bathymodiolus* symbiosis (∼3 μmol g^−1^ (wet weight of gill tissue) h^−1^) despite similar incubation H_2_ concentrations of 1800 ppm (Petersen *et al*., 2011). H_2_ consumption rates of free‐living and cultivated microorganisms are higher than the *O. algarvensis* symbiosis and range from ∼ 100 μmol g^−1^ (wet weight) h^−1^ to several mmol g^−1^ (wet weight) h^−1^ (V_max_) at H_2_ concentrations between 1 ppm and 20 000 ppm (Häring and Conrad, 1991; Klüber and Conrad, 1993; Perner *et al*., 2010). To adequately compare these rates to the H_2_ consumption rate of the *O. algarvensis* symbiosis the reaction kinetics of H_2_ oxidation would need to be characterized for the *O. algarvensis* symbiosis in future experiments. As discussed for CO, it is likely that the H_2_ oxidation rates of the *O. algarvensis* symbionts are considerably higher, because we calculated these rates based on whole worm wet weight, instead of the biomass of the two sulfate‐reducing symbionts predicted to be able to oxidize H_2_ (Kleiner *et al*., 2012b). If we assume that only both deltaproteobacterial symbionts oxidized H_2_ based on the observation that they possess hydrogenases (Kleiner *et al*., 2012b), and estimate their abundance at 10% of the total biomass of whole worms (Giere and Erseus, 2002; Ruehland *et al*., 2008), H_2_ oxidation rates would be 110 μmol g^−1^ (wet weight) h^−1^, which is in the range of those of free‐living and cultivated bacteria (Häring and Conrad, 1991; Klüber and Conrad, 1993; Perner *et al*., 2010).

The lag phase in H_2_ consumption during the first 40 h of incubation (Fig. 4) could be due to the oxic pre‐incubations of the worms and the relatively high oxygen concentrations at the beginning of the experiments (see Experimental Procedures). The expression of hydrogenases in anaerobic H_2_‐oxidizing microorganisms is generally only induced in the presence of H_2_ and repressed by oxygen (reviewed in Vignais and Billoud, 2007). It is thus likely that the symbionts did not have substantial amounts of hydrogenases at the beginning of the incubation and had to produce these after exposure to H_2_ and suboxic conditions in the incubation vessels. Once H_2_ consumption began, H_2_ was consumed down to 9 ppm in one incubation bottle, which corresponds to 5.4 nM H_2_ in solution (Fig. 4A), indicating that the *O. algarvensis* symbionts have hydrogenases that can take up H_2_ down to very low concentrations. This is in agreement with the metaproteome study of Kleiner and colleagues (2012b), which showed that the expressed uptake hydrogenases of the deltaproteobacterial symbionts are closely related to hydrogenases characterized as having a high‐affinity for H_2_ (Kleiner *et al*., 2012b).

Despite high H_2_ consumption rates of live *O. algarvensis* worms, none of the symbionts showed increased CO_2_ incorporation in the presence of H_2_ in the nanoSIMS analyses (Fig. 5). This suggests that H_2_ was not used as an energy source for autotrophic CO_2_ fixation by the hydrogenase‐possessing δ‐symbionts under the applied incubation conditions. The δ‐symbionts may have instead used the energy released by the oxidation of H_2_ for lithoheterotrophic growth as discussed for CO above.

### What are the sources of CO and H_2_ in the habitat of *O* *. algarvensis*?

In the photic zone of the ocean, CO is produced through non‐biological processes (abiotically) during the photochemical lysis of organic material. CO concentrations in the seawater above our study site were in the same low nanomolar range as those measured in other studies, and was most likely produced through photolytic processes (summarized and discussed in Tolli *et al*., 2006 and Moran and Miller, 2007). Surprisingly, there is currently no data on CO concentrations in marine sediments. In this study, we found CO concentrations in sediment pore waters that were twice as high as those in the overlying seawater, with the highest concentrations measured in the dead rhizome mats at sediment depths of 25 cm and more where photolysis of organic material is not possible (Figs 1 and 7). Accordingly, our results from incubations with different components from the *O. algarvensis* habitat showed that dead seagrass rhizomes incubated in the dark released considerable amounts of CO (Table 2). We therefore hypothesize that the large mats of dead seagrass rhizomes in the *O. algarvensis* habitat are a source of aphotically produced CO. Based on our observation that up to two orders of magnitude more CO was produced in the rhizome incubations in which biological activity was stopped with ZnCl_2_ than in the rhizome incubations without ZnCl_2_, we hypothesize that (1) CO production occurred abiotically, i.e. in the absence of live organisms, and (2) in the rhizome incubations without ZnCl_2_, CO was not only produced, but also consumed by microorganisms associated with the dead rhizomes. This hypothesis is supported by earlier studies that found abiotic production of CO from humic acids, phenolic compounds and decaying plant material in soils in the absence of light and the presence of an oxidant (Conrad and Seiler, 1980; 1982; 1985). These authors observed that CO production increased with increasing temperature, moisture content and alkaline conditions (higher pH), indicating that a thermochemical process is involved in the production of CO from decaying plant material in soils. The exact reaction mechanism behind this process remains unknown.

CO production from organic material could also explain the widespread presence of microorganisms with the genetic potential to oxidize CO in the aphotic zones of the ocean. Several metagenomic and one metatranscriptomic study found high frequencies of CODH genes used for CO oxidation in the Mediterranean, Pacific and Atlantic Oceans at water depths between 200 and 6000 m, but the CO source remained elusive (Martin‐Cuadrado *et al*., 2009; Quaiser *et al*., 2011; Smedile *et al*., 2013). We hypothesize that CO production from decaying organic matter is not limited to soils as described by Conrad and Seiler (1980; 1982; 1985) but could also explain CO production in the bathypelagic. This hypothesis is supported by studies showing ‘dark production’ of CO (i.e. not from photolytic processes) in coastal surface waters that can make up as much as 25% of the total CO production budget (Zhang *et al*., 2008; Day and Faloona, 2009).

In contrast to CO, none of the components from the *O. algarvensis* environment produced H_2_ under the aerobic conditions used in this study. Potential sources for the high H_2_ concentrations in the sediment pore waters at our collection site are the anaerobic oxidation of CO by carboxydotrophs that use protons as electron acceptors and thus release H_2_ (Kerby *et al*., 1995; Maness *et al*., 2005; Oelgeschlager and Rother, 2008), and microbial fermentation that would produce H_2_ as a by‐product (Schwartz and Friedrich, 2006). Difficult to explain are the unusually high H_2_ concentrations in the *O. algarvensis* sediments of 89–2147 nM because H_2_ concentrations in aquatic sediments are usually very low (<60 nM) because of rapid oxidation by free‐living H_2_ oxidizers (Goodwin *et al*., 1988; Novelli *et al*., 1988).

### Could CO production by dead seagrass rhizomes in the Mediterranean sediments support the *O* *. algarvensis* symbiosis?

CO concentrations in the pore waters of the *O. algarvensis* collection site (17–51 nM) correspond to atmospheric mixing ratios of 23–68 ppm. Such low concentrations (10–100 ppm) have been successfully used to incubate and isolate CO oxidizers from soils and seawater (Hendrickson and Kubiseski, 1991; Hardy and King, 2001; King, 2007; Weber and King, 2010).

In our incubations, *O. algarvensis* was also able to oxidize CO down to concentrations as low as 16 ppm (concentration at the end of one of the CO incubations) and down to 6.5 ± 35.8 nM in the incubations with ^13^CO (average and standard deviation for four parallel ^13^CO incubations), indicating that the symbionts would be able to take up CO at the concentrations measured in the worm's environment. Additionally, we consider it likely that the symbionts experience fluctuating conditions of CO and H_2_ supply and that CO concentrations may often be higher than those that we measured in the pore waters for two reasons. First, based on our data we hypothesize that CO flux in the sediments close to the dead rhizomes is very high despite the low concentrations measured in pore waters. We base this assumption on the fact that on average five times more CO was produced in dead rhizome incubations with ZnCl_2_ compared with without ZnCl_2_ (Table 2), suggesting that an active CO‐oxidizing microbial community rapidly oxidized the CO produced by the dead rhizomes. Second, the high variability in the measured pore water CO (and H_2_) concentrations indicate a high spatial variation and suggest that small pockets with high concentrations may exist in the sediment. However, our measuring method, which required large sample volumes, would not have allowed the detection of such fine spatial differences.

To estimate how many *O. algarvensis* worms could be sustained by using CO as sole energy source for growth, we calculated the amount of carbon that could be fixed with the CO produced in the *O. algarvensis* habitat assuming that (1) between 0.02 and 0.164 mol CO_2_ can be fixed autotrophically per mole CO oxidized using oxygen as terminal electron acceptor (Moersdorf *et al*., 1992); (2) an average *O. algarvensis* individual has a carbon content of 5 μmol; (3) 20 kg (dry weight) dead seagrass rhizome are buried in 1 m^2^ of sediment (rough estimate based on samples taken for incubations) which produce 36.5 mmol CO per year [using a conservative mean CO production value of 5 μmol kg^−1^ (dry weight) per day by dead rhizomes in their native state, Table 2]; and (4) CO production by dead rhizomes is constant. We calculated that up to 6 mmol carbon could be fixed per year and square meter using CO as an energy source, which is equal to the carbon content of 1200 worms. These estimates indicate that CO production from decaying plant material could be a significant energy source for microbial primary production in marine seagrass sediments in the Mediterranean Sea and possibly in other coastal regions with large amounts of decaying plant material.

## Experimental procedures

### Specimen collection and preparation for incubations

Worms were collected by scuba diving in October 2009 and October 2011 off Capo di Sant' Andrea, Elba in Italy (geographic position: 42°48′29.38′N, 10°8′31.57′E; 6–8 m of water depth). Only intact specimens were used in incubation experiments. Sexually mature worms that were identified as *O. ilvae*, a co‐occurring less abundant gutless oligochaete species, were sorted out and not used in the experiments (Giere and Erseus, 2002).

Internally stored sulfur in the γ1‐symbionts (Giere and Erseus, 2002) was removed by pre‐incubating all worms in large glass bowls containing 0.2 μM‐filtered oxic seawater and a thin (3 mm) layer of glass beads for a week. This pre‐treatment was necessary because the γ1‐symbionts use their stored sulfur for CO_2_ fixation, and this would have masked differences in CO_2_ fixation between treatments. After this pre‐incubation, the symbionts had lost most of their stored sulfur as determined by the change of worm colour from bright white to transparent and decreased CO_2_ fixation rates in test incubations without an external energy source (Fig. S3).

### Incubation experiments with ^13^ C‐labelled bicarbonate and CO, H_2_ or no external energy source

We compared uptake rates of ^13^C‐labelled bicarbonate in *O. algarvensis* worms under three conditions: (1) in the presence of CO, (2) in the presence of H_2_ and (3) in the absence of an externally added energy source. ASW with a salinity of 39‰ was prepared as previously described (Widdel and Bak, 1992) (Supporting Information). The pH of the ASW was adjusted to 7.5 corresponding to the conditions in the *O. algarvensis* habitat. ^13^C‐labelled NaHCO_3_^−^ was added to detect CO_2_ fixation in the symbionts.

Incubation bottles (serum bottles) were flushed with N_2_ gas prior to filling with 20 ml of ASW to create microaerobic conditions. Oxygen concentrations were measured at the end of the incubations with an amperometric microelectrode (Revsbech, 1989) and were 0.18 mM in the control bottles and 0.11 mM in the bottles containing live worms.

All incubations were run in triplicates with 35 live worms added to each serum bottle, whereas control incubations contained 35 dead worms (see Supporting Information), 5 μl of wash water (see Supporting Information) or only ASW. To start the incubation, either 80 μl of CO (purity level 3.7; Air Liquide, Düsseldorf, Germany) or 80 μl of H_2_ (purity level 5.0; Air Liquide) were injected into the headspace of the serum bottles; in the incubations without an external energy source nothing was added to the headspace. Serum bottles were stored at 22°C and gently tilted back and forth (18× per minute) to allow mixing and to reduce diffusion limitation. At given time points, subsamples from the headspace were taken with gas‐tight syringes and CO and H_2_ concentrations were measured with a Shimadzu GC‐8A gas chromatograph equipped with a Molecular Sieve 5A column and an RGD2 Reduction Gas Detector (Trace Analytical, Menlo Park, CA, USA) as described previously (Pohorelic *et al*., 2002). To stay within the linear range of the detector samples were diluted with pure nitrogen gas if needed. H_2_ and CO standards were produced from pure H_2_ (purity level 5.0; Air Liquide) and CO gas (purity level 3.7; Air Liquide) in pure nitrogen gas (purity level 5.0; Air Liquide). To control for potential instrument drift, standards were measured in each measurement run and used to calculate sample concentrations within each run (Table S2). After H_2_ was completely consumed, we added an additional 80 μl of H_2_ to the serum bottles containing live worms for a better time resolution of H_2_ consumption by the *O. algarvensis* symbiosis. At the end of the incubations, worms were processed for bulk tissue analyses and nanoSIMS (see below). CO and H_2_ consumption rates were calculated based on the linear CO consumption between 65 and 87 h (Fig. 2) and the linear H_2_ consumption during the last 17.5 h of the incubation using the average wet weight of one worm (0.5 mg) and the molar volume of an ideal gas at 22 °C (24.54 l mol^−1^).

### Bulk analysis of ^13^ C‐incorporation in whole worms

To determine incorporation of ^13^C‐labelled bicarbonate in whole worms, eight worms from each replicate were killed in 3 ml of ASW and 100 μl of aqueous zinc chloride (ZnCl_2_) solution (50% v/w). Worms were washed in ASW, rinsed briefly in 0.1% HCl to remove unfixed labelled bicarbonate, washed again in ASW, placed in tin cups and their wet weight measured. Dead worms from control incubations were treated the same way. Tin cups with worms were dried over night at 70°C and stored at room temperature until further processing. Carbon isotope composition of the worms was analysed using an automated elemental analyzer (Thermo Flash EA 1112) coupled to an isotopic ratio mass spectrometer (Thermo Delta Plus XP, Thermo Fisher Scientific) ^13^C isotope content in the worms was calculated as atom percent (AT% = ^13^C / (^12^C + ^13^C) × 100). Caffeine (Sigma‐Aldrich) was used as a standard for isotope calibration and quantification (Table S3).

### nanoSIMS analysis of ^13^ C‐incorporation into single symbiont cells

To determine the amount of ^13^C assimilated by each symbiont we analysed the carbon isotope composition of single symbiont cells using CARD‐FISH combined with nanoSIMS imaging (see Supporting Information for more details) (Musat *et al*., 2012; Polerecky *et al*., 2012).

### Incubation experiments with ^13^ CO

To investigate whether CO consumption was caused by oxidation to CO_2_ or by CO assimilation, labelled ^13^CO (99 AT% ^13^C, < 5 AT% ^18^O, Sigma‐Aldrich Cat. No. 388505) was added to incubations that were prepared as described above but with unlabelled ^12^C‐bicarbonate (Sigma‐Aldrich) added to the ASW. Incubations were done in 12 ml glass vials (Exetainers, Labco, High Wycombe, UK) without headspace, with four parallel incubations for each time point. Four worms were placed in an incubation vial and the incubation started by adding CO to a final concentration of 7 μM. At given time points, samples were killed by addition of ZnCl_2_ (50% w/v). Six milliliters of medium were transferred to 6 ml glass vials (Exetainers). Two milliliters of medium were taken with pure helium (He) gas (purity level 5.0; Air Liquide) and transferred to new He‐flushed 6 ml glass vials (Exetainers). For outgassing of CO_2_ into the headspace 0.2 ml of 85% phosphoric acid were injected. Two hundred fifty microlitres of the headspace were analysed with a gas chromatograph – isotope ratio mass spectrometer (VG Optima, Manchester, UK). Pure CO_2_ (purity 4.5; Air Liquide) was used as a standard for isotope calibration and quantification.

CO end‐point concentrations were measured after outgassing of CO into the 2 ml He headspace created in the 12 ml glass vials after the medium for the CO_2_ analyses was removed. The same measurement setup as for the CO and H_2_ worm incubations was used. The average CO concentration in the incubations with worms was blank corrected using the average CO concentration in control vessels without added CO to account for CO production by the rubber septa after killing of the worms.

### Measurement of CO and H_2_ concentrations in the worms' habitat

Sediment pore water and seawater above the sediment were collected at the worm collection site by scuba diving and measured as previously described (Kleiner *et al*., 2012b). Nine profiles were sampled within an area of approx. one hundred square metres at sediment depths of 15 cm, 25 cm and in the dead seagrass rhizomes (Fig. 1). Seawater samples were collected ∼5 cm above the sediment surface. H_2_ and CO concentrations were measured with an RGA3 reduction gas analyser (Trace Analytical) using ultra pure nitrogen (purity level 5.0; SOL s.p.a., Monza, Italy) as carrier gas. To control for potential instrument drift, standards were measured in each measurement run and used to calculate sample concentrations within each run (Table S2).

Eight blanks with ddH_2_O were created, processed and measured in the same way as the pore water samples. Average CO concentrations (3.1 nM) and H_2_ concentrations (21.5 nM) in blanks were used for blank correction of pore water and seawater concentrations. All concentrations in this study are given as blank‐corrected values.

### CO and H_2_ production of different components from the *O* *. algarvensis* habitat

To identify the sources of CO and H_2_ in the *O. algarvensis* environment, we incubated dead seagrass rhizome, sediment and seawater from six different locations at the worm collection site (Fig. 1). From each location, 70–100 g (wet weight) rhizome material (collected from the layer of dead rhizome mats at ≥ 25 cm sediment depth), 250 ml of sediment or unfiltered seawater were added to 0.5 l Schott bottles. We filled bottles completely with sterile filtered seawater, closed them with rubber septa and then withdrew 20 ml of seawater using a syringe to create a headspace. Bottles with ddH_2_O were used as controls. Bottles were incubated in a water bath set at 23°C for ∼ 3.5 h in the dark to avoid photochemical CO production (King and Weber, 2007). Ten minutes before measuring H_2_ and CO concentrations, bottles were shaken thoroughly to allow produced H_2_ and CO gas to equilibrate with the headspace. H_2_ and CO were measured using the same setup as for the pore water samples (see above). Rhizome samples and sediment samples were rinsed in freshwater and dried after the incubation to determine their dry weight.

To distinguish biotic from abiotic CO and H_2_ production, samples were either incubated in their native state or amended with ZnCl_2_ (50% w/v) to a final concentration of 61 mM to stop biological activity. The pH was adjusted to the pH of the native seawater (pH 7.7–7.8) in ZnCl_2_‐amended samples. The minimum inhibitory concentration of ZnCl_2_ for microbial activity is about 1 mM (Winslow and Haywood, 1931; He *et al*., 2002; Choi *et al*., 2010). We are therefore confident that the 61‐fold higher concentration in our experiments efficiently stopped most biological activity.

## Supporting information


**Fig. S1.** Epifluorescence images of *O. algarvensis* symbionts on a filter. Symbiont cells are the same as in Fig. 6. Images in the left and right columns show in the top row the composite CARD‐FISH signals of all fluorescence channels, followed in the second row by the epifluorescence images of symbiont cells with the sulfur‐oxidizing symbionts targeted by the gammaproteobacterial probe (Gam42a) in red. The third row shows epifluorescence images of all symbiont cells targeted by the general eubacterial probe (EUB338I‐III) in green and the fourth row epifluorescence images of the general DNA stain DAPI in blue.
**Fig. S2.** Comparison of the aerobic form I CODH operons in known CO oxidizers and the γ3‐symbiont. While the metagenomic sequences of the γ3‐symbiont CODH genes used in the metaproteomic study of the *O. algarvensis* symbionts were fragmented and distributed among two genome contigs (Woyke *et al*., 2006; Kleiner *et al*., 2012), we recovered the complete and uninterrupted CODH operon of the γ3‐symbiont as part of our 2012 Community Sequencing Project (CSP) with the US Department of Energy Joint Genome Institute (see Acknowledgments). The bottom three microorganisms are known to oxidize CO at very low concentrations (<1000 ppm). Bold letters highlight the functional subunits of the CODH (coxMSL), while the other genes are accessory proteins (e.g. coxDEF). Genes shown in white do not belong to the CODH operon and genes labeled with # indicate a gene found in many CODH operons, but not in the *O. carboxidovorans* genome. This gene (#) is annotated as ‘CTP:molybdopterin cytidylytransferase’ according to RAST. Genes are not drawn to scale. The CSP 2012 contig is available upon request.
**Fig. S3.** Comparison of ^13^C isotope content in white and pale worms after incubation with ^13^C‐labeled bicarbonate, nitrate and oxygen, but no additional external energy source for 36 h. The γ1‐symbionts in white worms contain large amounts of stored elemental sulfur, which they use for CO_2_ fixation under oxic conditions (Giere, 2006). In pale worms, the sulfur stores of the γ1‐symbionts are reduced or depleted leading to less CO_2_ fixation. Pale worms were obtained by oxic pre‐incubations (see Experimental procedures). Mean values and standard deviations of five (for the white worms) and three (for the pale worms) independent incubations are shown. ^13^C isotope content values are given in atomic percentage (AT%=(^13^C/(^12^C+^13^C)x100).
**Table S1.** ^13^C isotope fraction data for individual regions of interest (ROIs).
**Table S2.** Values for individual measurements of CO and H_2_ standards.
**Table S3.** Results from bulk analyses of whole worms.
