# Supplementary material for: Use of carbon monoxide and hydrogen by a bacteria–animal symbiosis from seagrass sediments
Source: Environ Microbiol. 2015 Jul 23;17(12):5023–35. doi: 10.1111/1462-2920.12912 (PMC4744751; doi:10.1111/1462-2920.12912)
Supplement: Supplementary file 1 — Fig. S1. Epifluorescence images of O. algarvensis symbionts on a filter. Symbiont cells are the same as in Fig. 6. Images in the left and right columns show in the top row the composite CARD‐FISH signals of all fluorescence channels, followed in the second row by the epifluorescence images of symbiont cells with the sulfur‐oxidizing symbionts targeted by the gammaproteobacterial probe (Gam42a) in red. The third row shows epifluorescence images of all symbiont cells targeted by the general eubacterial probe (EUB338I‐III) in green and the fourth row epifluorescence images of the general DNA stain DAPI in blue. Fig. S2. Comparison of the aerobic form I CODH operons in known CO oxidizers and the γ3‐symbiont. While the metagenomic sequences of the γ3‐symbiont CODH genes used in the metaproteomic study of the O. algarvensis symbionts were fragmented and distributed among two genome contigs (Woyke et al., 2006; Kleiner et al., 2012), we recovered the complete and uninterrupted CODH operon of the γ3‐symbiont as part of our 2012 Community Sequencing Project (CSP) with the US Department of Energy Joint Genome Institute (see Acknowledgments). The bottom three microorganisms are known to oxidize CO at very low concentrations (<1000 ppm). Bold letters highlight the functional subunits of the CODH (coxMSL), while the other genes are accessory proteins (e.g. coxDEF). Genes shown in white do not belong to the CODH operon and genes labeled with # indicate a gene found in many CODH operons, but not in the O. carboxidovorans genome. This gene (#) is annotated as ‘CTP:molybdopterin cytidylytransferase’ according to RAST. Genes are not drawn to scale. The CSP 2012 contig is available upon request. Fig. S3. Comparison of 13C isotope content in white and pale worms after incubation with 13C‐labeled bicarbonate, nitrate and oxygen, but no additional external energy source for 36 h. The γ1‐symbionts in white worms contain large amounts of stored elemental sulfur, which they use for CO2 fixa [file EMI-17-5023-s001.zip › EMI_12912-supp-0001-20150510_CO-H2_Suppl_1stRev_FINAL.pdf]

Supporting Information

Use of carbon monoxide and hydrogen by a bacteria-animal symbiosis from seagrass sediments

Manuel Kleiner, Cecilia Wentrup, Thomas Holler, Gaute Lavik, Jens Harder, Christian Lott, Sten Littmann, Marcel M. M. Kuypers, and Nicole Dubilier

Experimental Procedures

Incubation experiments with ^{13}C-labeled bicarbonate and CO, H_2 or no added energy source

Artificial seawater (ASW) was prepared as follows (final concentrations): 0.8 mM KBr, 8.1 mM KCl, 10 mM $CaCl_2$ x $2H_2O$, 27.9 mM $MgCl_2$ x $6H_2O$, 27.6 mM $MgSO_4$ x $7H_2O$, 451.2 mM NaCl, 1 mM $NaNO_3^-$, 0.5 mM NH_4Cl, 0.2 mM KH_2PO_4, 5.9 mM ^{13}C-labeled $NaHCO_3^-$, 0.02 µM Na_2WO_4 x $2H_2O$, 0.02 µM Na_2SeO_3 x $5H_2O$ and trace elements (modified after Widdel and Bak, 1992) (all chemicals were purchased from Sigma-Aldrich, Munich, Germany). Nitrate was added to ensure metabolic activity of the γ3-symbiont based on the observation that proteins for nitrate respiration were abundant in this symbiont, while oxygen respiration proteins were absent (Kleiner et al., 2012). 20 ml of ASW were filled into sterile 59 ml serum bottles that contained a layer of glass beads to accommodate the thigmotactic behavior of the worms (2:1 (v/v); 0.75 – 1 mm (ROTH, Karlsruhe, Germany): 0.4 – 0.6 mm (B. Braun Biotech International, Melsungen, Germany)). Serum bottles were closed with gas-tight butyl rubber stoppers.

Controls with dead worms and wash water

Worms for control incubations were killed with 4% (v/v) formaldehyde for 15 min at 4 °C and washed three times in ASW for 5 min before adding them to the incubation bottles. Wash water controls consisted of sterile ASW in which live worms were washed before the incubations and were used as a control for potential free-living microorganisms or sediment particles from the worm surface.

Nanoscale secondary ion mass spectrometry (nanoSIMS) analysis of ^{13}C-incorporation into single symbiont cells

Three *O. algarvensis* worms from each treatment were homogenized after the incubations and fixed in 2% paraformaldehyde for 2 hours at 4 °C. Symbiont cells were immobilized on gold-palladium coated polycarbonate filters (0.22 µm) by filtration of worm homogenates as previously described (Musat et al., 2008). Filters were washed by filtering 10 ml of sterile ASW to reduce host debris. Using this approach most of the host debris was removed, however, larger host tissue pieces, mitochondria and nuclei stayed on the filters. To identify the different symbionts and distinguish them from host debris we used catalyzed reporter deposition fluorescence *in situ* hybridizations (CARD-FISH) (Pernthaler et al., 2002) with the general bacterial probe EUB338 (Daims et al., 1999), the general gammaproteobacterial probe GAM42a (Manz et al., 1996) and the γ3-symbiont specific probe (Ruehland et al., 2008). Epifluorescence images of the hybridized symbionts on the filters were taken before nanoSIMS analysis and regions of interest were marked using a laser micro-dissecting microscope (LMD, Leica, Germany) to enable their localization during nanoSIMS analyses (Polerecky et al., 2012). Regions of interest had to meet the following criteria if they were to be analyzed with nanoSIMS: Symbionts had to have a clear CARD-FISH signal and be

clearly separated from any other symbiont cells or host debris. Symbiont clumps, mitochondria and other unidentified tissue were excluded from nanoSIMS analysis. An additional quality control was possible through the sulfur and phosphorus signals obtained in the nanoSIMS analysis, which were distinct for each symbiont species. Fluorescence signals of the symbiont cells were processed by blind deconvolution in AutoQuant (Media Cybernetics, Bethesda, USA). Symbionts were distinguished based on probe signal and their known size and morphology (Giere and Erseus, 2002; Ruehland et al., 2008).

The nanoSIMS analyses were performed with a NanoSIMS 50L (Cameca, Gennevilliers, France). The areas of interest were scanned with a Cs^+ primary ion beam with a beam current between 0.8 and 1.2 pA. We used five electron multipliers in parallel to record secondary ion images of $^{12}C^-$, $^{13}C^-$, $^{12}C^{14}N^-$, $^{31}P^-$, and $^{32}S^-$ from at least 30 single cells per symbiont and treatment (Fig. 5) with raster sizes between 8 to 40 µm and resolutions of 256 x 256 pixels or 512 x 512 pixels. We used a dwell time of 1 ms per pixel. The only exception were γ3-symbiont cells incubated with H_2 for which we only analyzed 19 cells from two worms due to their low abundance on the filter from the third worm (Fig. 5). We were unable to measure sufficient numbers of the spirochete symbiont for statistical analyses, because this symbiont is so thin (100-200 nm) that cells were completely destroyed during the pre-sputter process with the primary ion beam of the nanoSIMS.

Image and data processing were performed using the Look@NanoSIMS software (Polerecky et al., 2012). To evaluate data quality and to check for potential drift in instrument performance we used the carbon signal emitted by the polycarbonate filter on which cells were immobilized. For this, two regions of interest (ROIs) were drawn in each image in areas without biological matter. We then checked that no differences in ^{13}C isotope content between the two ROIs, indicative of instrument drift, were seen. Images with inconsistent

values for background ROIs were excluded from further analysis. The ^{13}C isotope content data for background ROIs is included in the supplemental table containing the ^{13}C isotope content data for all ROIs (Table S1).

^{13}C isotope content of single symbiont cells was calculated as atom percent (AT% = ($^{13}C/(^{12}C+^{13}C)x100$). For comparison of ^{13}C-content in specific symbiont species between different treatments homogeneity of variance was tested using the Levene's test. Depending on the outcome of the Levene's test, significant differences between means were tested using either the ANOVA test or the Kruskal-Wallis test implemented in Look@NanoSIMS (Polerecky et al., 2012).

Table S1. ^{13}C isotope fraction data for individual regions of interest (ROIs)

Please see separate Excel file.

Table S2. Values for individual measurements of CO and H_2 standards

RGD2 Reduction Gas Detector used for worm incubation experiments

		Replicates (Peak area in mV*min)					
	Standard	Area A	Area B	Area C	AVG Area	SD Area	SD in %
Incubation Day 1	50 ppm CO	303	308	300	304	3.8	1.2
Incubation Day 2	50 ppm CO	307	306	316	310	5.3	1.7
Incubation Day 3	50 ppm CO	301	310	310	307	5.1	1.7
Incubation Day 4	50 ppm CO	298	324	292	305	17.1	5.6
Incubation Day 5	50 ppm CO	298	311	313	307	7.8	2.5
Incubation Day 6	50 ppm CO	286	289	no data	288	2.2	0.8
Incubation Day 1	200 ppm H2	55	54	52	54	1.6	2.9
Incubation Day 2	200 ppm H2	48	49	49	49	0.9	1.8
Incubation Day 3	200 ppm H2	53	52	47	51	3.0	6.0
Incubation Day 5	200 ppm H2	47	50	45	47	2.9	6.1
Incubation Day 5	200 ppm H2	46	55	50	50	4.2	8.3
Incubation Day 5	200 ppm H2	45	49	47	47	1.9	4.1
Incubation Day 6	200 ppm H2	43	46	44	44	1.4	3.2
Incubation Day 6	200 ppm H2	41	42	42	42	0.7	1.7

RGA3 Reduction Gas Analyzer used for pore water concentrations and sediment component incubations

		Replicates (Peak area in mV*min)				
Standard		Area A	Area B	AVG Area	SD Area	SD in %
Incubation Day 1	10 ppm CO	23.18	23.51	23.35	0.23	1.0
Incubation Day 2	10 ppm CO	24.01	23.96	23.99	0.04	0.2
Incubation Day 3	10 ppm CO	23.47	22.02	22.75	1.03	4.5
Incubation Day 4	10 ppm CO	25.89	23.15	24.52	1.94	7.9
Incubation Day 1	100 ppm H2	16.05	16.01	16.03	0.02	0.2
Incubation Day 2	100 ppm H2	18.66	18.57	18.62	0.07	0.4
Incubation Day 3	100 ppm H2	14.31	14.48	14.40	0.12	0.8
Incubation Day 4	100 ppm H2	17.50	17.04	17.27	0.33	1.9
Pore water	10 ppm CO	32.29	32.78	32.54	0.34	1.1
Pore water	10 ppm H2	2.05	2.16	2.11	0.08	3.6

Table S3. Results from bulk analyses of whole worms

Run 1	Identifier 1	AT% ^{13}C
	Caffeine	1.065963
	Caffeine	1.065819
	Caffeine	1.06589
	Caffeine	1.065706
	Caffeine	1.066085
	Caffeine	1.06577
	Caffeine	1.065641
	Worms_13CO2_a	1.355234
	Worms_13CO2_b	1.293376
	Worms_13CO2_c	1.259979
	Worms_13CO2/CO_a	1.281095
	Worms_13CO2/CO_b	1.27354
	Worms_13CO2/CO_c	1.298852
	Worms_13CO2/H2_a	1.296299
	Worms_13CO2/H2_b	1.256732
	Worms_13CO2/H2_c	1.331806

Run 2	Identifier 1	AT% ^{13}C
	Caffeine	1.06642
	Caffeine	1.066348
	Caffeine	1.066194
	Caffeine	1.066292
	Caffeine	1.070564
	Dead worms	1.069968
	Dead worms	1.072227
	Dead worms	1.073281
	Dead worms	1.07138
	Dead worms	1.07356
	Dead worms	1.072551

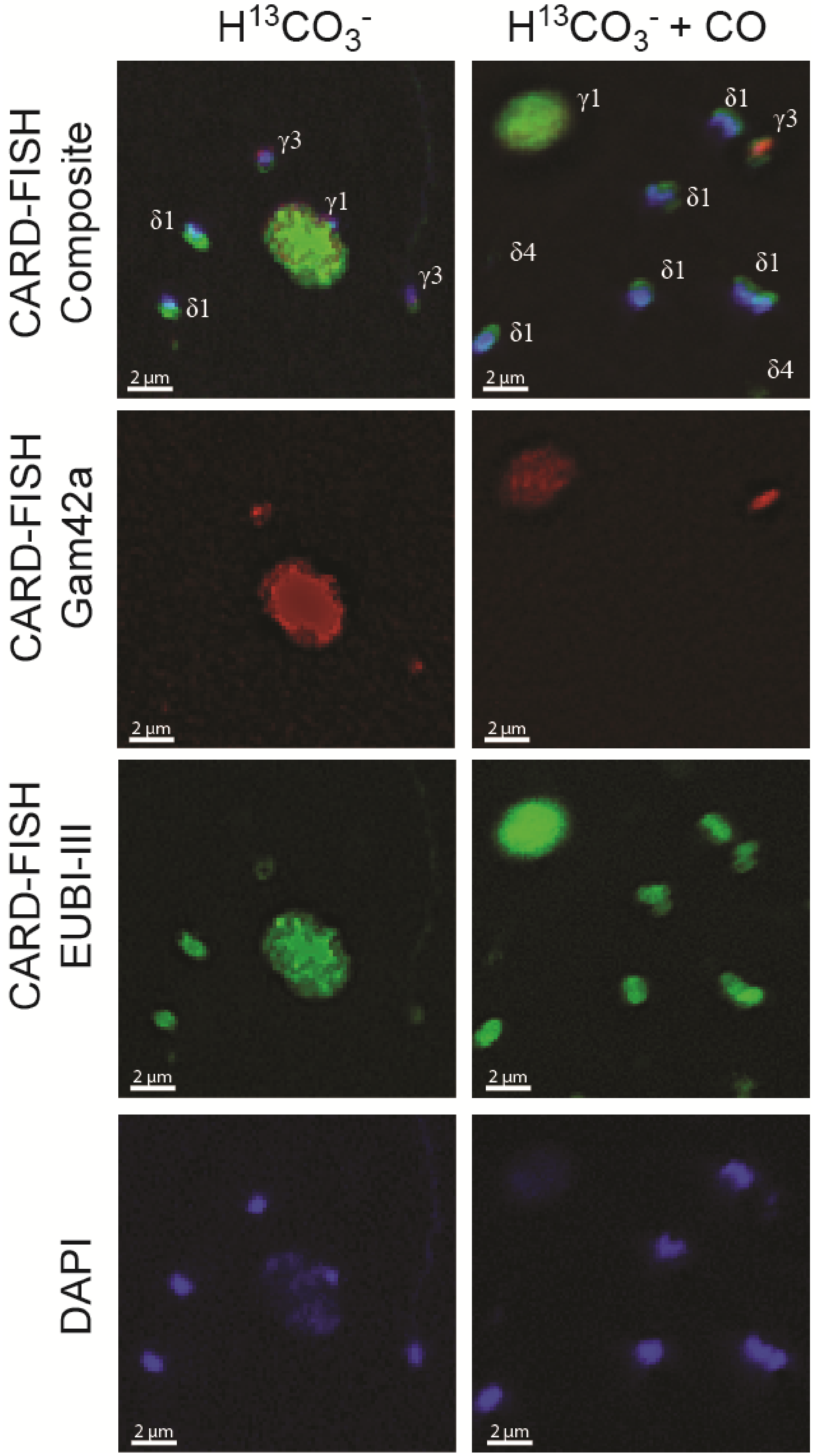

H13CO3-
H13CO3- + CO
CARD-FISH Composite
CARD-FISH Gam42a
CARD-FISH EUBI-III
DAPI
γ3
γ1
δ1
δ1
γ3
γ1
δ1
γ3
δ1
δ4
δ1
δ1
δ1
δ4
2 µm

Figure S1: Epifluorescence images of *O. algarvensis* symbionts on a filter. Symbiont cells are the same as in Fig. 6. Images in the left and right columns show in the top row the composite CARD-FISH signals of all fluorescence channels, followed in the second row by the epifluorescence images of symbiont cells with the sulfur-oxidizing symbionts targeted by the gammaproteobacterial probe (Gam42a) in red. The third row shows epifluorescence images of all symbiont cells targeted by the general eubacterial probe (EUB338I-III) in green and the fourth row epifluorescence images of the general DNA stain DAPI in blue.

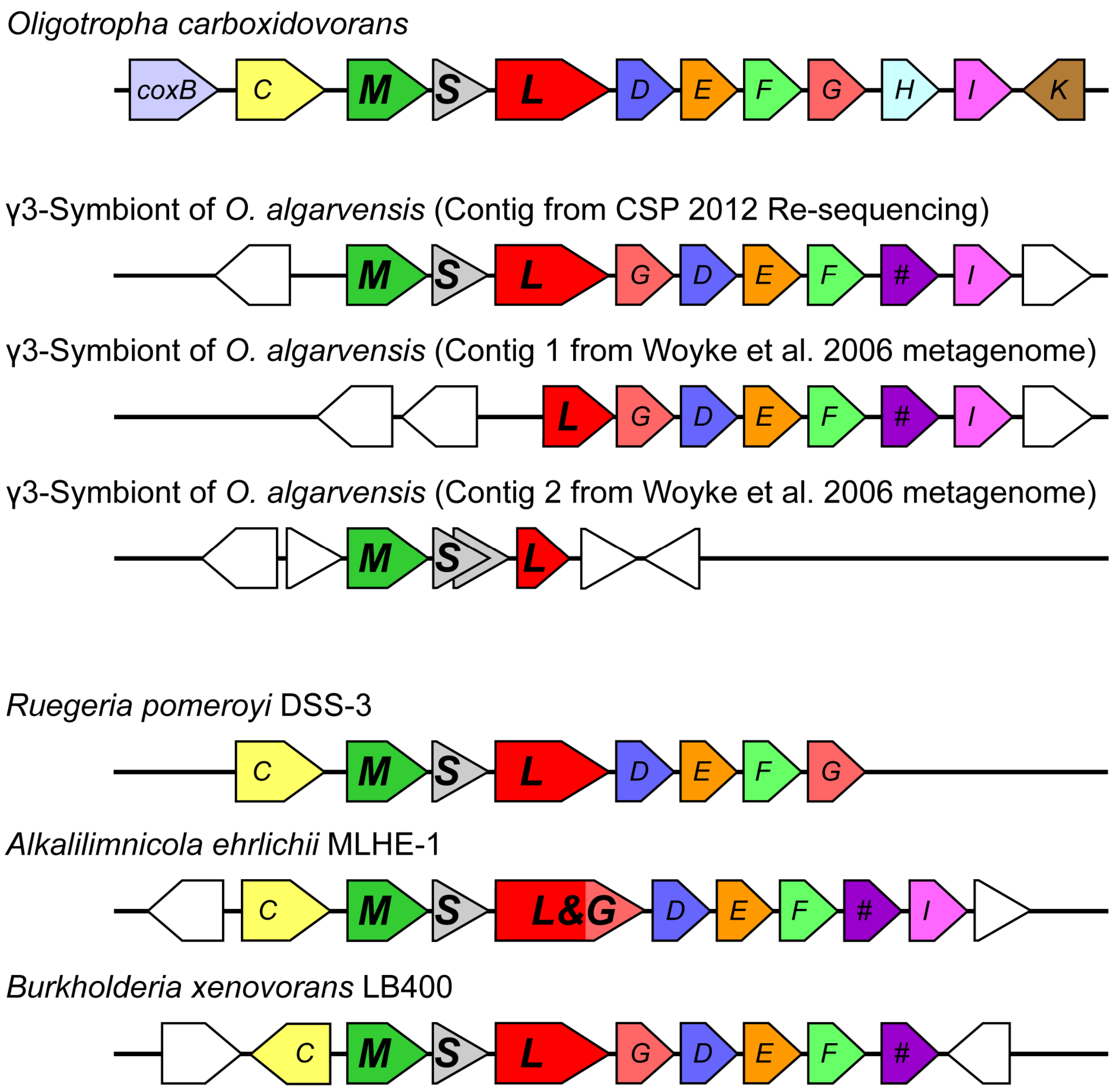
Oligotropha carboxidovorans
coxB C M S L D E F G H I K
γ3-Symbiont of O. algarvensis (Contig from CSP 2012 Re-sequencing)
M S L G D E F # I
γ3-Symbiont of O. algarvensis (Contig 1 from Woyke et al. 2006 metagenome)
L G D E F # I
γ3-Symbiont of O. algarvensis (Contig 2 from Woyke et al. 2006 metagenome)
M S L
Ruegeria pomeroyi DSS-3
C M S L D E F G
Alkalilimnicola ehrlichii MLHE-1
C M S L&G D E F # I
Burkholderia xenovorans LB400
C M S L G D E F #

Figure S2: Comparison of the aerobic form I CODH operons in known CO oxidizers and the γ3-symbiont. While the metagenomic sequences of the γ3-symbiont CODH genes used in the metaproteomic study of the *O. algarvensis* symbionts were fragmented and distributed among two genome contigs (Woyke et al., 2006; Kleiner et al., 2012), we recovered the complete and uninterrupted CODH operon of the γ3-symbiont as part of our 2012 Community Sequencing Project (CSP) with the US Department of Energy Joint Genome Institute (see Acknowledgements). The bottom three microorganisms are known to oxidize CO at very low concentrations (< 1000 ppm). Bold letters highlight the functional subunits of the CODH (coxMSL), while the other genes are accessory proteins (e.g. coxDEF). Genes shown in white do not belong to the CODH operon and genes labeled with # indicate a gene found in many CODH operons, but not in the *O. carboxidovorans* genome. This gene (#) is annotated as 'CTP:molybdopterin cytidylytransferase' according to RAST. Genes are not drawn to scale. The CSP 2012 contig is available upon request.

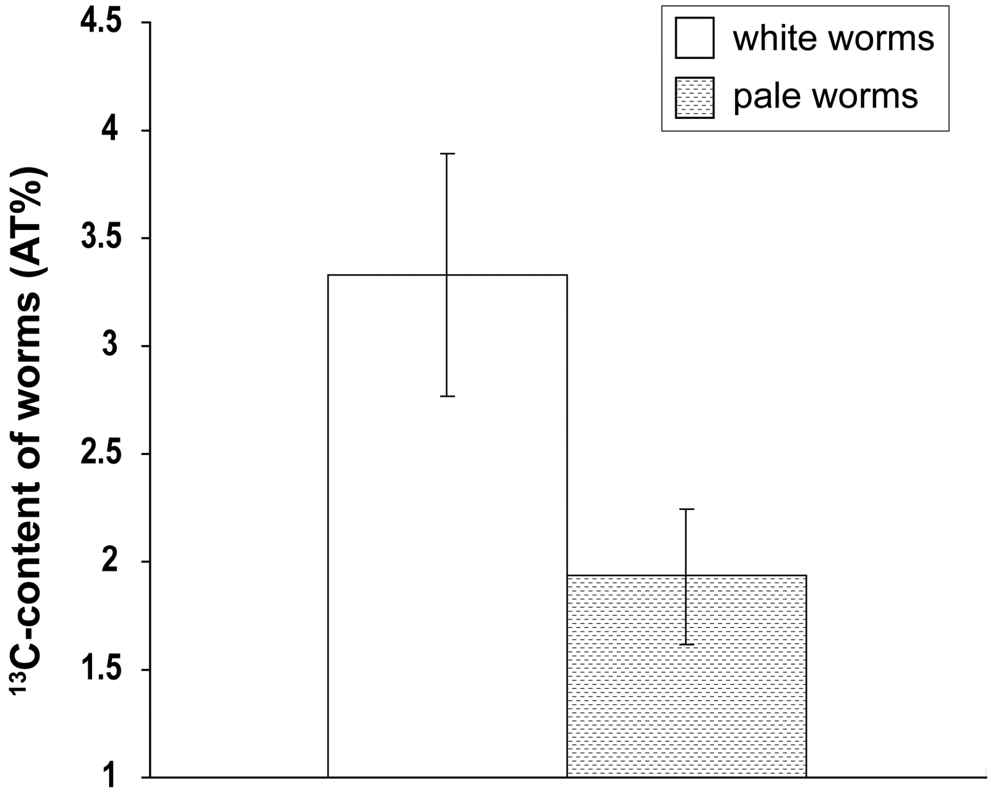

Figure S3: Comparison of ^{13}C isotope content in white and pale worms after incubation with ^{13}C-labeled bicarbonate, nitrate and oxygen, but no additional external energy source for 36 hours. The γ1-symbionts in white worms contain large amounts of stored elemental sulfur, which they use for CO_2 fixation under oxic conditions (Giere, 2006). In pale worms, the sulfur stores of the γ1-symbionts are reduced or depleted leading to less CO_2 fixation. Pale worms were obtained by oxic pre-incubations (see Material and Methods). Mean values and standard deviations of five (for the white worms) and three (for the pale worms) independent incubations are shown. ^{13}C isotope content values are given in atomic percentage (AT%= ($^{13}C/(^{12}C+^{13}C)$x100).

References

Daims, H., Bruhl, A., Amann, R., Schleifer, K.H., and Wagner, M. (1999) The domain-specific probe EUB338 is insufficient for the detection of all Bacteria: Development and evaluation of a more comprehensive probe set. *Syst Appl Microbiol* **22**: 434-444.

Giere, O. (2006) Ecology and biology of marine oligochaeta - an inventory rather than another review. *Hydrobiologia* **564**: 103-116.

Giere, O., and Erseus, C. (2002) Taxonomy and new bacterial symbioses of gutless marine Tubificidae (Annelida, Oligochaeta) from the Island of Elba (Italy). *Org Divers Evol* **2**: 289–297.

Kleiner, M., Wentrup, C., Lott, C., Teeling, H., Wetzel, S., Young, J. et al. (2012) Metaproteomics of a gutless marine worm and its symbiotic microbial community reveal unusual pathways for carbon and energy use. *Proc Natl Acad Sci U S A* **109**: E1173-1182.

Manz, W., Amann, R., Ludwig, W., Vancanneyt, M., and Schleifer, K.H. (1996) Application of a suite of 16S rRNA-specific oligonucleotide probes designed to investigate bacteria of the phylum cytophaga-flavobacter-bacteroides in the natural environment. *Microbiology-Uk* **142**: 1097-1106.

Musat, N., Halm, H., Winterholler, B., Hoppe, P., Peduzzi, S., Hillion, F. et al. (2008) A single-cell view on the ecophysiology of anaerobic phototrophic bacteria. *Proc Natl Acad Sci U S A* **105**: 17861-17866.

Pernthaler, A., Pernthaler, J., and Amann, R. (2002) Fluorescence in situ hybridization and catalyzed reporter deposition for the identification of marine bacteria. *Appl Environ Microbiol* **68**: 3094-3101.

Polerecky, L., Adam, B., Milucka, J., Musat, N., Vagner, T., and Kuypers, M.M. (2012) Look@NanoSIMS-a tool for the analysis of nanoSIMS data in environmental microbiology. *Environ Microbiol* **14**: 1009-1023.

Ruehland, C., Blazejak, A., Lott, C., Loy, A., Erseus, C., and Dubilier, N. (2008) Multiple bacterial symbionts in two species of co-occurring gutless oligochaete worms from Mediterranean seagrass sediments. *Environ Microbiol* **10**: 3404-3416.

Widdel, F., and Bak, F. (1992) Gram-negative mesophilic sulfate-reducing bacteria. In *The Prokaryotes*. A. Balows, H.G.T., M. Dworkin, W. Harder, K-H. Schleifer (ed): Springer, New York, pp. 3352-3378.

Woyke, T., Teeling, H., Ivanova, N.N., Huntemann, M., Richter, M., Gloeckner, F.O. et al. (2006) Symbiosis insights through metagenomic analysis of a microbial consortium. *Nature* **443**: 950-955.
